# CERAPP: Collaborative Estrogen Receptor Activity Prediction Project

**DOI:** 10.1289/ehp.1510267

**Published:** 2016-02-23

**Authors:** Kamel Mansouri, Ahmed Abdelaziz, Aleksandra Rybacka, Alessandra Roncaglioni, Alexander Tropsha, Alexandre Varnek, Alexey Zakharov, Andrew Worth, Ann M. Richard, Christopher M. Grulke, Daniela Trisciuzzi, Denis Fourches, Dragos Horvath, Emilio Benfenati, Eugene Muratov, Eva Bay Wedebye, Francesca Grisoni, Giuseppe F. Mangiatordi, Giuseppina M. Incisivo, Huixiao Hong, Hui W. Ng, Igor V. Tetko, Ilya Balabin, Jayaram Kancherla, Jie Shen, Julien Burton, Marc Nicklaus, Matteo Cassotti, Nikolai G. Nikolov, Orazio Nicolotti, Patrik L. Andersson, Qingda Zang, Regina Politi, Richard D. Beger, Roberto Todeschini, Ruili Huang, Sherif Farag, Sine A. Rosenberg, Svetoslav Slavov, Xin Hu, Richard S. Judson

**Affiliations:** 1 National Center for Computational Toxicology, U.S. Environmental Protection Agency, Research Triangle Park, North Carolina, USA; 2 Oak Ridge Institute for Science and Education, Oak Ridge, Tennessee, USA; 3 Institute of Structural Biology, Helmholtz Zentrum Muenchen-German Research Center for Environmental Health (GmbH), Neuherberg, Germany; 4 Chemistry Department, Umeå University, Umeå, Sweden; 5 Environmental Chemistry and Toxicology Laboratory, IRCCS (Istituto di Ricovero e Cura a Carattere Scientifico)-Istituto di Ricerche Farmacologiche Mario Negri, Milan, Italy; 6 Laboratory for Molecular Modeling, University of North Carolina at Chapel Hill, Chapel Hill, North Carolina, USA; 7 Laboratoire de Chemoinformatique, University of Strasbourg, Strasbourg, France; 8 National Cancer Institute, National Institutes of Health (NIH), Department of Health and Human Services (DHHS), Bethesda, Maryland, USA; 9 Institute for Health and Consumer Protection (IHCP), Joint Research Centre of the European Commission in Ispra, Ispra, Italy; 10 Department of Pharmacy-Drug Sciences, University of Bari, Bari, Italy; 11 Division of Toxicology and Risk Assessment, National Food Institute, Technical University of Denmark, Copenhagen, Denmark; 12 Milano Chemometrics and QSAR Research Group, University of Milano-Bicocca, Milan, Italy; 13 Division of Bioinformatics and Biostatistics, National Center for Toxicological Research, U.S. Food and Drug Administration (USDA), Jefferson, Arizona, USA; 14 BigChem GmbH, Neuherberg, Germany; 15 High Performance Computing, Lockheed Martin, Research Triangle Park, North Carolina, USA; 16 Research Institute for Fragrance Materials, Inc., Woodcliff Lake, New Jersey, USA; 17 Integrated Laboratory Systems, Inc., Research Triangle Park, North Carolina, USA; 18 Division of Systems Biology, National Center for Toxicological Research, USDA, Jefferson, Arizona, USA; 19 National Center for Advancing Translational Sciences, NIH, DHHS, Bethesda, Maryland, USA

## Abstract

**Background:**

Humans are exposed to thousands of man-made chemicals in the environment. Some chemicals mimic natural endocrine hormones and, thus, have the potential to be endocrine disruptors. Most of these chemicals have never been tested for their ability to interact with the estrogen receptor (ER). Risk assessors need tools to prioritize chemicals for evaluation in costly in vivo tests, for instance, within the U.S. EPA Endocrine Disruptor Screening Program.

**Objectives:**

We describe a large-scale modeling project called CERAPP (Collaborative Estrogen Receptor Activity Prediction Project) and demonstrate the efficacy of using predictive computational models trained on high-throughput screening data to evaluate thousands of chemicals for ER-related activity and prioritize them for further testing.

**Methods:**

CERAPP combined multiple models developed in collaboration with 17 groups in the United States and Europe to predict ER activity of a common set of 32,464 chemical structures. Quantitative structure–activity relationship models and docking approaches were employed, mostly using a common training set of 1,677 chemical structures provided by the U.S. EPA, to build a total of 40 categorical and 8 continuous models for binding, agonist, and antagonist ER activity. All predictions were evaluated on a set of 7,522 chemicals curated from the literature. To overcome the limitations of single models, a consensus was built by weighting models on scores based on their evaluated accuracies.

**Results:**

Individual model scores ranged from 0.69 to 0.85, showing high prediction reliabilities. Out of the 32,464 chemicals, the consensus model predicted 4,001 chemicals (12.3%) as high priority actives and 6,742 potential actives (20.8%) to be considered for further testing.

**Conclusion:**

This project demonstrated the possibility to screen large libraries of chemicals using a consensus of different in silico approaches. This concept will be applied in future projects related to other end points.

**Citation:**

Mansouri K, Abdelaziz A, Rybacka A, Roncaglioni A, Tropsha A, Varnek A, Zakharov A, Worth A, Richard AM, Grulke CM, Trisciuzzi D, Fourches D, Horvath D, Benfenati E, Muratov E, Wedebye EB, Grisoni F, Mangiatordi GF, Incisivo GM, Hong H, Ng HW, Tetko IV, Balabin I, Kancherla J, Shen J, Burton J, Nicklaus M, Cassotti M, Nikolov NG, Nicolotti O, Andersson PL, Zang Q, Politi R, Beger RD, Todeschini R, Huang R, Farag S, Rosenberg SA, Slavov S, Hu X, Judson RS. 2016. CERAPP: Collaborative Estrogen Receptor Activity Prediction Project. Environ Health Perspect 124:1023–1033; http://dx.doi.org/10.1289/ehp.1510267

## Introduction

There are tens of thousands of natural and synthetic chemical substances to which humans and wildlife are exposed (Dionisio et al. 2015; Egeghy et al. 2012; Judson et al. 2009). A subset of these compounds may disrupt normal functioning of the endocrine system and cause health hazards to both humans and ecological species (Birnbaum and Fenton 2003; Diamanti-Kandarakis et al. 2009; Mahoney and Padmanabhan 2010; UNEP and WHO 2013). Endocrine-disrupting chemicals (EDCs) can mimic or interfere with natural hormones and alter their mechanisms of action at the receptor level, as well as interfere with the synthesis, transport, and metabolism of endogenous hormones (Diamanti-Kandarakis et al. 2009). Exposure to EDCs can lead to adverse health effects involving developmental, neurological, reproductive, metabolic, cardiovascular, and immune systems in humans and wildlife (Colborn et al. 1993; Davis et al. 1993; Diamanti-Kandarakis et al. 2009).

The estrogen receptor (ER) is one of the most extensively studied targets related to the effects of EDCs (Mueller and Korach 2001; Shanle and Xu 2011). This concern about estrogen-like activity of man-made chemicals is because of their potential for negatively affecting reproductive function (Hileman 1994; Kavlock et al. 1996). The emergence of concerns about EDCs has resulted in regulations requiring assessment of chemicals for estrogenic activity [Adler et al. 2011; U.S. Environmental Protection Agency (EPA) 1996; U.S. Food and Drug Administration (FDA) 1996]. There are numerous *in vitro* and *in vivo* protocols to identify potential endocrine pathway-mediated effects of chemicals, including interactions with hormone receptors (Jacobs et al. 2008; Rotroff et al. 2013; Shanle and Xu 2011; Sung et al. 2012). However, experimental testing of chemicals is expensive and time-consuming and currently impractical for application to the vast number of synthetic chemicals in use. Consequently, toxicological data and especially estrogenic activity data are available only for a limited number of compounds (Cohen Hubal et al. 2010; Egeghy et al. 2012; Judson et al. 2009).

The use of *in silico* approaches, such as quantitative structure–activity relationships (QSARs), is an alternative to bridge the lack of knowledge about chemicals when little or no experimental data are available. These structure-based methods are particularly appealing for their ability to predict toxicologically relevant end points quickly and at low cost (Muster et al. 2008; Vedani and Smiesko 2009). QSARs have been promoted and their use recognized since the pioneering work of Hansch in the 1960s (Fujita et al. 1964; Hansch et al. 1962; Hansch and Deutsch 1966). The conceptual basis of QSARs is that chemicals with similar structures are hypothesized to exhibit similar behavior in living organisms. Thus, it should be possible to predict biological activity of new chemicals based on published experimental data. Several guidance documents to develop these modeling techniques are available in the literature (Dearden et al. 2009; Worth et al. 2005).

Recently, *in vitro* high-throughput screening (HTS) assays have emerged and become a viable tool for large-scale chemical testing (Judson et al. 2011; Kavlock and Dix 2010; Wetmore et al. 2012). HTS generates substantial amounts of data that can be used as a knowledge base to correlate chemical structures to their biological activities. Thus, QSARs can identify key structural characteristics in active chemicals and can use them to virtually screen large chemical libraries. Although there is concern about the overall accuracy of a QSAR model to predict the true activity of a particular chemical, accuracy can be high enough to use the results for prioritizing chemicals that are worth subjecting to experimental testing.

With the increasing number of new substances submitted to the U.S. EPA and the European Chemicals Agency for registration (~ 1,500 chemicals every year), there is a need to prioritize chemicals to speed up the process and lower the overall costs of testing (U.S. EPA 2015). The Toxicology Testing in the 21st Century (Tox21) collaboration and the U.S. EPA’s Toxicity ForeCaster (ToxCast™)projects are screening thousands of chemicals in HTS *in vitro* assays for a broad range of targets (Dix et al. 2007; Judson et al. 2010; Martin et al. 2010). Relevant to this paper, these two projects have in common ~ 1,800 chemicals tested in a battery of 18 ER-related assays (Huang et al. 2014; Judson et al. 2015).

This paper describes the results of the Collaborative Estrogen Receptor Activity Prediction Project (CERAPP), which was organized by the National Center for Computational Toxicology at the U.S. EPA. The aim of the project was to use ToxCast™/Tox21 ER HTS assay data to develop and optimize predictive computational models, and to use their predictions to prioritize a large chemical universe of 32,464 unique chemical structures for further testing. Seventeen research groups from the United States and Europe participated in this project. These groups submitted 40 categorical models and 8 continuous models using different QSAR and structure-based approaches. Most of the newly developed models used a training set consisting of 1,677 chemicals, each assigned a potency score quantifying their ER agonist, antagonist, and binding activities, obtained from a computational network model that integrates data from 18 diverse ER HTS assays (Judson et al. 2015). All models were evaluated and weighted based on their prediction accuracy scores (including sensitivity and specificity) using ToxCast™/Tox21 HTS data, as well as an evaluation data set collected from different literature sources. To overcome the limitations of single models, all predictions were combined into a *consensus* model that classified the chemicals into active/inactive binders, agonists, and antagonists and provided estimates of their potency level relative to known reference chemicals.

## Materials and Methods

### Participants and Project Planning

The 17 international research groups that participated in this project are listed in alphabetic order in Table S1. The goals of the project, outlined in Table S2, were achieved in multiple steps, including chemical structure curation, experimental data preparation from the literature, modeling and prediction, model evaluation, consensus strategy development, and consensus modeling. Each step was assigned to a subgroup of participants according to their interests and areas of expertise.

### Data Sets

***Provided training set.*** The data that were suggested to be used by the participants as a training set to develop and optimize their models was derived from ToxCast™ and Tox21 programs (Dix et al. 2007; Huang et al. 2014; Judson et al. 2010). Concentration-response data from a collection of 18 *in vitro* HTS assays exploring multiple sites in the mammalian ER pathway were generated for 1,812 chemicals (Judson et al. 2015; U.S. EPA 2014c). This chemical library included 45 reference ER agonists and antagonists (including negatives), as well as a wide array of commercial chemicals with known estrogen-like activity (Judson et al. 2015). A mathematical model was developed to integrate the *in vitro* data and calculate an area under the curve (AUC) score, ranging from 0 to 1, which is roughly proportional to the consensus AC50 value across the active assays (Judson et al. 2015). A given chemical was considered active if its agonist or antagonist score was higher than 0.01. In order to reduce the number of potential false positives this threshold can be increased to 0.1.

***Prediction set.*** We identified > 50,000 chemicals [at the level of Chemical Abstracts Service Registry Number (CASRN)] for use in this project as a virtual screening library to be prioritized for further testing and regulatory purposes. This set was intended to include a large fraction of all man-made chemicals to which humans may be exposed. These chemicals were collected from different sources with significant overlap and cover a variety of classes, including consumer products, food additives, and human and veterinary drugs. The following list includes the sources used in this project:

Chemicals with documented use, and therefore, with exposure potential (~ 43,000). Available in the U.S. EPA chemical product categories database (CPCat), which is part of the Aggregated Computational Toxicology Resource (ACToR) system (Dionisio et al. 2015; Judson et al. 2008, 2012; U.S. EPA 2014a).
The Distributed Structure-Searchable Toxicity (DSSTox) (U.S. EPA 2014b). A list of ~ 15,000 curated chemical structures from multiple inventories of environmental interest. In particular, structures for all of the ToxCast™ and Tox21 chemicals are included.
The Canadian Domestic Substances list (DSL) (Environment Canada 2012). A compiled list of all substances thought to be in commercial use in Canada (~ 24,000 chemicals). Thus, it includes chemicals with potential human or ecological exposure.
The Endocrine Disruption Screening Program (EDSP) universe of ~ 10,000 chemicals. The U.S. EPA’s EDSP is required to test certain chemicals for their potential for endocrine disruption (U.S. EPA 2014d).
A list of ~ 15,000 chemicals used as training and test sets for the different models implemented in the U.S. EPA’s Estimation Program Interface (EPI Suite™) to predict physico-chemical properties (U.S. EPA 2014e).

This virtual chemical library has undergone stringent chemical structure processing and normalization for use in the QSAR modeling study (see “Chemical Structure Curation”) and made available for download on ToxCast™ Data web site under CERAPP data (https://www3.epa.gov/research/COMPTOX/CERAPP_files.html, PredictionSet.zip) (U.S. EPA 2016), is intended to be employed for a large number of other QSAR modeling projects, not just those focused on endocrine-related targets.

***Experimental evaluation set.*** A large volume of estrogen-related experimental data has accumulated in the literature over the past two decades. The information on the estrogenic activity of chemicals was mined and curated to serve as a validation set for predictions of the different models. For this purpose, *in vitro* experimental data were collected from different overlapping sources, including the U.S. EPA’s HTS assays, online databases, and other data sets used by participants to train models:

HTS data from Tox21 project consisting of ~ 8,000 chemicals evaluated in four assays (Attene-Ramos et al. 2013; Collins et al. 2008; Huang et al. 2014; Shukla et al. 2010; Tice et al. 2013), extending beyond the 1,677 used in the training set.
The U.S. FDA Estrogenic Activity Database (EADB), which consists of literature derived ER data for ~ 8,000 chemicals (Shen et al. 2013).
Estrogenic data for ~ 2,000 chemicals from the METI (Ministry of Economy, Trade and Industry, Japan) database (METI 2002).
Estrogenic data for ~ 2,000 chemicals from ChEMBL database (Gaulton et al. 2012).

The full data set consisted of > 60,000 entries, including binding, agonist, and antagonist information for ~ 15,000 unique chemical structures. For the purpose of this project, this data set was cleaned and made more consistent by removing *in vivo* data, cytotoxicity information, and all ambiguous entries (missing values, undefined/nonstandard end points, and unclear units). Only 7,547 chemical structures from the experimental evaluation set that overlapped with the CERAPP prediction set, for a total of 44,641 entries, were kept and made available for download on the U.S. EPA ToxCast™ Data web site (https://www3.epa.gov/research/COMPTOX/CERAPP_files.html, EvaluationSet.zip) (U.S. EPA 2016). The non-CERAPP chemicals were excluded from the evaluation set (see “Chemical Structure Curation” section). Then, all data entries were categorized into three assay classes: (*a*) binding, (*b*) reporter gene/transactivation, or (*c*) cell proliferation. The training set end point to model is the ER model AUC that parallels the corresponding individual assay AC_50_ values, and therefore all units for activities in the experimental data set were converted to μM to have approximately equivalent concentration–response values for the evaluation set. Chemicals with cell proliferation assays were considered as actives if they exceeded an arbitrary threshold of 125% proliferation. For entries where testing concentrations were reported in the assay name field, those values were converted to μM and considered as the AC_50_ value if the compound was reported as active. All inactive compounds were arbitrarily assigned an AC_50_ value of 1 M.

### Chemical Structure Curation

Chemical structures collected from different public sources contained many duplicates, and inconsistencies in the molecular structures. Hence, a structure curation process was carried out to derive a unique set of QSAR-ready structures. All participating groups then used this consistent set of structures for both training and prediction steps. It should be noted that each group likely employed different descriptor calculation software, which could effectively alter structures in some cases. Several different curation approaches were combined into a unique procedure used for this project (Fourches et al. 2010; Wedebye et al. 2013). The free and open-source data-mining environment KNIME (Konstanz Information Miner) was selected to design a curation workflow to process all structures and provide consistent training and prediction sets (Berthold et al. 2007). The workflow performed a series of curation steps:

1. The original files containing structures in different formats were parsed, checked for valences, and for the integrity of the required structural information to render the molecules. Invalid entries were corrected by retrieving a new structure from online databases using web services [PubChem (NIH 2015), ChemSpider (Royal Society of Chemistry 2015)] or removed if ambiguous.
2. The first filter was applied to check for the presence of carbon atoms and remove inorganic compounds.
3. The structures were desalted, and inorganic counterions were removed.
4. The second filter, based on molecular weight, was applied and chemicals exceeding a threshold of 1,000 g/mol were removed to speed up molecular descriptor calculations and model calibration.
5. Valid QSAR modeling practice requires all chemicals to be structurally consistent by converting tautomers to unique representations. Thus, a series of transformations was applied on the structures to standardize nitro and azide mesomers, keto-enol tautomers, enamine-imine tautomers, ynol-ketene, and other conversions (ChemAxon 2014; Reusch 2013; Sitzmann et al. 2010).
6. These transformations were followed by neutralizing the charged structures, when possible, and removing the stereochemistry information.
7. Explicit hydrogen atoms were added, and structures were aromatized according to Hückel’s rules implemented in KNIME (Berthold et al. 2007).
8. The duplicates were removed using the IUPAC (International Union of Pure and Applied Chemistry) InChI (International Chemical Identifier) codes because these are unequivocal identifiers.
9. The final filter was applied to remove chemicals containing metals that often cause problems in molecular descriptor calculations.

Both training and prediction sets were processed by the same structure curation workflow. At the end of this procedure, 32,464 unique structures—the 32 K set—remained in the prediction set and 1,677 in the training set. These two data sets are made available for download in structure data file (SDF) format on the U.S. EPA ToxCast™ Data web site (https://www3.epa.gov/research/COMPTOX/CERAPP_files.html, TrainingSet.zip and PredictionSet.zip) (U.S. EPA 2016). The identity of these chemicals (name, CASRN) was not provided to the participating modeling groups during the modeling process.

### Modeling Approaches

The participant groups adopted different approaches and used several software programs (proprietary or open-source [commercial or free]) to calibrate categorical and continuous models to the training data (Table 1). A categorical model is one that provides an active/inactive call for each chemical, whereas a continuous model provides a prediction of the potency (in μM) for each active chemical. Models were developed using both well-known and innovative methods including partial least-squares (PLS) (Ståhle and Wold 1987; Wold et al. 2001), partial least-squares discriminant analysis (PLS-DA) (Frank and Friedman 1993; Nouwen et al. 1997), decision forest (DF) (Hong et al. 2005, 2004; Tong et al. 2003; Xie et al. 2005), three-dimensional (3D) quantitative spectral data–activity relationship (QSDAR) (Beger et al. 2001; Beger and Wilkes 2001; Slavov et al. 2013), support vector machines (SVM) (Cristianini and Shawe-Taylor 2000), *k* nearest neighbors (kNN) (Cover and Hart 1967; Kowalski and Bender 1972), associative artificial neural networks (ASNN) (Tetko 2002a, 2002b), PASS algorithm derived from Naïve Bayes classifier (Poroikov et al. 2000), self-consistent regression with radial basis function interpolation (RBF-SCR) (Zakharov et al. 2014), OCHEM machine learning methods (Tetko et al. 2014), docking and *consensus* of different approaches (Horvath et al. 2014; Ng et al. 2014; Sushko et al. 2011). The set of 1,677 chemicals provided by the U.S. EPA was used by more than 90% of the participating groups as a training set to fit their models (Judson et al. 2015), but some pre-existing models were also used that had been trained using other data sets from the literature such as METI (2002). In addition, each group performed its own analysis to select the appropriate chemicals to be considered as a training set according to their particular modeling procedure. For descriptor calculation and docking procedures, some of the programs used were LeadScope (Roberts et al. 2000), PaDEL-Descriptor (Yap 2011), QikProp (version 3.4, http://www.schrodinger.com/QikProp/), multilevel and quantitative neighborhoods of atoms (MNA, QNA) used by GUSAR and PASS (Filimonov et al. 2009; Poroikov et al. 2000), DRAGON (Talete srl 2012), Mold2 (Hong et al. 2008, 2012), GLIDE (version 6.5, http://www.schrodinger.com/Glide), AutoDock (Goodsell et al. 1996), ISIDA (Varnek et al. 2008), and other fingerprint generators. Some of the participants applied feature selection techniques, such as genetic algorithms (GAs) (Davi 1991) and random forest (RF) (Breiman 2001). These techniques were applied after calculating descriptors to reduce collinearity and variable dimensionality to keep only the most informative descriptors in the models.

**Table 1:** Methods adopted by the participant groups (alphabetic order) in the modeling procedure.

Model name	Calibration method	Descriptors software/type	Training set (No. of chemicals)	Predictions type
DTU	PLS/fragments	Leadscope	METI (595,481)/ToxCast^™^ (1,422)	Categorical
EPA_NCCT	GA + PLSDA	PADEL	ToxCast^™^ (1,529)	Categorical
FDA_NCTR_DBB (Ng et al. 2014)	DF	Mold2	ToxCast^™^ (1,677)	Categorical
FDA_NCTR_DSB	PLS	3D-SDAR	ToxCast^™^ (1019)	Categorical
ILS_EPA (Zang et al. 2013)	SVM + RF	Qikprop	ToxCast^™^ (1,677)	Categorical
IRCCS_CART (Roncaglioni et al. 2008)	CART-VEGA	2D descriptors	METI (806)	Categorical
IRCCS_Ruleset	Ruleset	SMARTS	ToxCast^™^ (1,529)	Categorical
JRC_Ispra (Poroikov et al. 2000)	PASS	MNA	—	Categorical
Lockheed Martin	kNN	Fingerprints	ToxCast^™^ (1,677)	Categorical + continuous
NIH_NCATS	Docking	AutoDock score	—	Categorical
NIH_NCI_GUSAR (Filimonov et al. 2009)	RBF-SCR	MNA, QNA	ToxCast^™^ (1,677)	Categorical
NIH_NCI_PASS (Poroikov et al. 2000)	PASS	MNA	ToxCast^™^ (1,677)	Categorical
OCHEM (2015)	*Consensus*	11 Descriptor types	ToxCast^™^(1,660)	Categorical + continuous
RIFM	SVM	Fingerprints	ToxCast^™^ (1,677)	Categorical
Umeå (Rybacka et al. 2015)	ASNN	DRAGON	METI + (Kuiper et al. 1997; Taha et al. 2010)	Categorical
UNC_MML	SVM+RF	DRAGON	ToxCast^™^ (120)	Categorical
UNIBA (Trisciuzzi et al. 2015)	Docking	GLIDE score	ToxCast^™^ (1,677)	Categorical
UNIMIB	kNN	DRAGON + fingerprints	ToxCast^™^ (1,677)	Categorical
UNISTRA (Horvath et al. 2014)	SVM	ISIDA	ToxCast^™^ (1,529)	Categorical + continuous
Predictions type: A categorical model is one that provides an active/inactive call for each chemical, whereas a continuous model provides a prediction of the potency (in μM) for each active chemical. Calibration methods: PLS (partial least-squares), PLS-DA (partial least-squares discriminant analysis), SVM (support vector machines), RF (random forest), DF (Decision forest), kNN (*k* nearest neighbors), ASNN (associative artificial neural networks), PASS (algorithm derived from Naïve Bayes classifier), RBF-SCR (self-consistent regression with radial basis function interpolation).				

### Evaluation Procedure for the Categorical and Continuous Models

All molecular structures of chemicals collected for the evaluation set from the different sources were curated and standardized using the previously described KNIME workflow (Table S2, step 2). All data used as the evaluation set for categorical and continuous models are available on the U.S. EPA ToxCast™ web site (https://www3.epa.gov/research/COMPTOX/CERAPP_files.html, EvaluationSet.zip) (U.S. EPA 2016).

Standard InChI codes were generated in KNIME and used to identify the chemicals. Data-mining tools available in the KNIME environment were used to concatenate and unify the different information fields from the different sources (CASRN, chemical name, original structure, standardized structure, InChI code, assay name, assay class, protein subtype, species, end point name, end point value, end point unit, and literature reference). Although ToxCast™ chemicals were used in the training sets of many models, they were not removed from the evaluation set to investigate how the predictions will perform on the literature data because there are differences between the AUC values and the literature data and because the sources from which the evaluation set was collected were not fully verified (we cannot assume that all cytotoxicity information was already fully cleaned).

***Evaluation set for categorical models.*** An important issue with the literature-derived evaluation set was the inconsistency of the results from different sources. To minimize this, the available entries for each chemical structure were grouped into binders, agonists, and antagonists. The results were then categorized into active and inactive classes using all available literature sources by applying three rules:

1. If, for a specific chemical within one of the three classes (binding, agonist, and antagonist), the disagreement among the different sources exceeded 20% (e.g., two sources indicating active agonist and three indicating inactive agonist), that chemical was removed from the evaluation data set of that specific class.
2. If a chemical was an active agonist or antagonist, it also was considered as an active binder if the information was not available.
3. If a chemical was an inactive agonist and inactive antagonist, it was considered also as nonbinder if the information was not available.

This procedure resulted in a total of 7,522 unique chemical structures with activity data to be used for evaluation of the categorical models (Table 2). It is also available for download on the U.S. EPA ToxCast™ web site (https://www3.epa.gov/research/COMPTOX/CERAPP_files.html, EvaluationSet.zip) (U.S. EPA 2016).

**Table 2:** Evaluation set for binary categorical models. Distribution of the number of active and inactive chemicals within the three different classes: binding, agonists and antagonists.

Class/activity	Active	Inactive	Total
Binding	1,982	5,301	7,283
Agonist	350	5,969	6,319
Antagonist	284	6,255	6,539
Total	2,017	7,024	7,522
The classification into actives and inactives is based on a consensus between the literature data sources that were in agreement.			

***Evaluation set for continuous models.*** For active chemicals with available quantitative information from concentration-response assays, the log_10_-median of the literature values was calculated. Only entries with equivalent end points were considered (e.g., PC50 and EC50). This resulted in 7,253 unique chemicals with quantitative information (Table 3 and https://www3.epa.gov/research/COMPTOX/CERAPP_files.html, EvaluationSet.zip) (U.S. EPA 2016). To reduce the variability that increased with the disparate literature sources, the chemicals with quantitative information were categorized into five potency activity classes: inactive, very weak, weak, moderate, and strong. These five classes were used to evaluate the quantitative predictions. A list of 36 known active and inactive reference chemicals was used for calibrating the mapping from quantitative potency values to the activity potency classes (Judson et al. 2015). These same chemicals were used to validate the mathematical model used to generate the AUC values for the training set. The following thresholds were applied to the concentration–response values:

**Table 3:** Evaluation set for quantitative models. Distribution of the number of chemicals in the five potency levels within the three different classes (binding, agonists, and antagonists), classifications based on average scores.

Class/activity	Inactive	Very weak	Weak	Moderate	Strong	Total
Binding	5,042	685	894	72	77	6,770
Agonist	5,892	19	179	31	42	6,163
Antagonist	6,221	76	188	10	10	6,505
Total	6,892	702	916	81	93	7,253
The classification of the chemicals in the five potency levels is based on the concentration responses from the literature sources that were in agreement.

Strong: Activity concentration below 0.09 μM.
Moderate: Activity concentration between 0.09 and 0.18 μM.
Weak: Activity concentration between 0.18 and 20 μM.
Very Weak: Activity concentration between 20 and 800 μM.
Inactive: Activity concentration higher than 800 μM.

The five classes were assigned scores from 0 (inactive) to 1 (strong) with 0.25 increments. Then, for each chemical, the arithmetic mean of the scores of the merged entries from different literature sources was calculated. A new class was assigned to the merged entries according to the following thresholds.

Strong: Average score > 0.75
Moderate: 0.5 < Average score between ≤ 0.75
Weak: 0.25 < Average score ≤ 0.5
Very weak: 0 < Average score ≤ 0.25
Inactive: Average score = 0

The number of entries in each class for binding, agonist, and antagonist are summarized in Table 3.

***Evaluation procedure.*** This section is focused on the categorical models for their high number compared to the continuous models. The procedure used to evaluate the predictions of the participant groups was based on the categorical and continuous experimental data from ToxCast™ and the evaluation set from the literature. All continuous and categorical models for binding, agonist, and antagonist were evaluated separately on the overlap between their predicted chemicals and the following sets of chemicals (Table S3).

Chemicals in the U.S. EPA’s ToxCast™ data set (*n* = 1,529 chemicals after excluding those in the ambiguous AUC range of 0.01–0.1).
All chemicals in the full literature data (all literature sources combined).
All chemicals with at least two literature sources.
All chemicals from the literature data excluding the very weak actives.
Chemicals within the applicability domain (AD) of each model (if provided).
Chemicals remaining after applying the previous three filters in steps 3, 4, and 5 to reduce ambiguous predictions (single literature source, very weak actives, and predictions outside the AD).

To evaluate the models on different criteria, we first determined the sensitivity (fraction of accurately predicted actives out of all actives), specificity (fraction of accurately predicted inactives out of all inactives), and balanced accuracy (BA; average of sensitivity and specificity) for each subgroup of chemicals according to each model. We then used BA values to derive two summary scores for each model, as described below.

***Score_1.*** Evaluation includes BA of each of the six steps weighted by the fraction of predicted chemicals of the same step, as well as the fraction of the predicted chemicals out of the full prediction set. This score (Equation 1) favors models with a wider AD and those predicting a maximum number of chemicals.


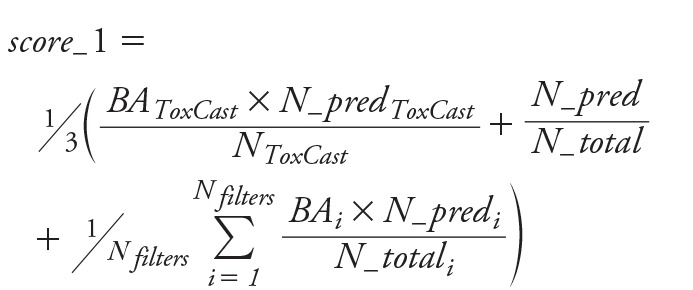
 [1]

where *BA* is balanced accuracy, *N_pred* is the number of predicted chemicals by a specific model, *N_total* is the total number of chemicals in the prediction set, *N_filters_* represents the number of five filters applied to the evaluation set chemicals and *i* the steps 2, 3, 4, 5, and 6.

***Score_2.*** Evaluation includes the BA of the model on the ToxCast™ data and the BA on the unambiguous chemicals (i.e., the subgroup of chemicals from the literature that remained after excluding chemicals with only one literature source, very weak chemicals, and chemicals outside of the AD, if provided). It favors models that focused on predicting more accurately but potentially with a narrower AD (Equation 2).

*score_*2 = ^1^/_2_ (*BA_ToxCast_* + *BA_all filters_*) [2]

The quantitative predictions were evaluated as categorical models (using the BA) of the five classes after converting the numerical predictions to potency classes as defined earlier (see “Evaluation set for continuous models” section). Scores of the continuous models were calculated using Equation 2.

### *Consensus* Modeling

The *consensus* predictions were generated for binders, agonists, and antagonists separately. For each chemical, we derived the average Score 2 value for all categorical models that predicted the chemical as active, and the average Score 2 value for all categorical models that predicted the chemical as inactive; we used the higher of the two averages to classify the chemical as active or inactive. Models that did not provide a prediction for the chemical in question were not included when deriving the average scores. We used Score 2 to derive the consensus classifications because its value for individual models is not penalized for the number of chemicals not predicted by the model. Also, the concordance among models on both active and inactive classes was calculated for each chemical as the fraction of models with positive and negative prediction, respectively.

Considering only the models that provided predictions, the sum of the concordance among models for actives and inactives is equal to 1. Because most models were associated with comparable scores, the average score used to classify chemicals was mostly in agreement with model concordance (i.e., the average score for actives is high when the concordance among the models with active predictions is high and vice versa). The few exceptions were noticed when model concordance was around 0.5, which means only one or two models were driving the classification.

For continuous predictions, the weight (*w*) for each chemical *i* was calculated from the scores (Equation 3):


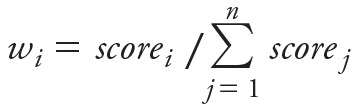
 [3]

where *n* is the total number of models that provided predictions for the chemical *i*, and *score_j_* is the score of the *j*th model predicting chemical *i*.

Next, the *consensus* potency level *C_i_* of each chemical was determined using the predicted potency classes *P_j_* of the *n* available models and their corresponding weights *w* as follows (Equation 4):


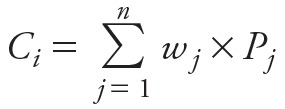
 [4]

## Results and Discussion

### Models and Evaluation

A total of 48 models were received from the 17 participant groups. Each group provided at least 1 categorical model for binding. Only 8 groups built models for agonists, and 6 groups built models for antagonists. The limited number of models for agonists and antagonists was the result of the low number of actives, which caused the training set to be highly unbalanced. The total number of models in each class (Table 1; see also Tables S3 and S5) was *a*) binding models: 21 categorical and 3 continuous, *b*) agonist models: 11 categorical and 3 continuous, and *c*) antagonist models: 8 categorical and 2 continuous.

The participating groups provided predictions for uneven fractions of the 32 k set. AD information on model predictions was provided by only six groups. All predictions for the individual models are provided on the U.S. EPA ToxCast™ web site (https://www3.epa.gov/research/COMPTOX/CERAPP_files.html, Models.zip) (U.S. EPA 2016).

The same evaluation procedure was applied to all models following the previously described steps. Note that some models were built using training sets other than what was provided in CERAPP and that these alternative training sets were not all publicly available. Hence, none of the training set chemicals were excluded from the evaluation sets (Table 1). Each model was evaluated on the overlap between the predicted chemicals and the two previously mentioned data sets: ToxCast™ data and the evaluation set collected from the literature. The evaluation results for categorical models are summarized in Table S3. The detailed statistics, including sensitivity and specificity, are provided in Table S4.

Most compounds were predicted as inactives and the models seemed to be more in agreement in predicting inactives than active compounds. Only 757 chemicals (2.33%) are predicted as actives by more than 75% of binding models. The agreement among the binding models for the 32 k set of the prediction set is illustrated in Figure S1.

Most categorical models (binding, agonist, and antagonist) are associated with high balanced accuracies on the ToxCast™ data (> 0.8), with no clear difference between models that used it as a training set and those that did not (see Table S3). However, for the evaluation set from the literature, the BA is clearly lower for all models (< 0.7). Nonetheless, the BA increased after removing chemicals with only one source from the literature data. This result could mean that this first filter (i.e., removing chemicals with limited information in the literature for being either positive or negative) reduced the uncertainty in the experimental data from the literature. This is in agreement with related studies showing that the results of QSAR models may change depending on the robustness of the experimental values (Steinmetz et al. 2014). The second filter (i.e., removing very weak actives) also increased the BA, which suggests that the literature data may contain a number of false positives. Alternatively, the *in vitro* assays used by ToxCast™/Tox21 only test chemicals up to 100 μM, so very weak chemicals may not be picked up by these assays and some of the literature reports may have tested chemicals up to much higher concentrations.

Finally, removing predictions outside the AD did not show improvement of the BA of the categorical models (see Table S3). This is in agreement with literature sources showing that predictions outside the AD are not always less accurate than those within its limits (Sahigara et al. 2012). The performance of most models showed a clear improvement of 0.05 to 0.1 on the BA after applying all the filters on the literature data to keep only the unambiguous chemicals. We believe that this effectively reduced the uncertainty of the literature sources. This step also highlighted differences between ToxCast™ and the literature data and confirmed the existence of uncertainty in the literature data. Uncertainty and data discordance was also reported in literature review of *in vivo* uterotrophic bioassays (Kleinstreuer et al. 2015).

The calculated scores for categorical models (see Table S3) take into consideration the whole prediction set (Score_1) and the accuracy of the model on its most reliable predictions (Score_2). The models that provided predictions for the whole or most of the 32 k set of chemicals, and had wide ADs, showed high Score_1 values (Umeå 0.82, OCHEM 0.83). Whereas models with predictions for smaller fractions of the prediction set and narrow AD showed better Score_2 values (UNIMIB_2 0.85, UNIBA 0.80). NIH_NCI_GUSAR (0.87 and 0.84) and FDA_NCTR_DBB (0.88 and 0.84) showed the highest values for both Score_1 and Score_2. Part of the differences among model scores could result from the uncertainty in the literature data.

The BAs of all antagonist models was low compared with binding and agonist models (see Table S3). This may be due to the highly unbalanced training set with a low number of active antagonist chemicals. Additionally, antagonism activity (in either ToxCast™ or the literature) can be confounded with cytotoxicity because antagonist transactivation assays are loss-of-signal assays.

The predictions of all continuous models were first converted to five classes using the list of reference chemicals as described in the evaluation set section (see “Evaluation set for continuous models” section). The predictions were then evaluated on the ToxCast™ data and the literature data to calculate the average of BA of the different evaluation steps as the score of each model (see Table S5). All models showed high BA on ToxCast™ data and relatively good BA on the evaluation set.

### Consensus *Model*

The *consensus* predictions were first evaluated on the ToxCast™ data and then on the evaluation set from the literature. The total number of predicted active binders was 2,661 out of the 32 k set of chemicals (8.2%) based on the method described in the “Materials and Methods” section “*Consensus* Modeling.”

Confusion matrices (Table 4) and prediction statistics (Table 5) revealed a clear accuracy difference between the categorical *consensus* for binding on the ToxCast™ data and on the evaluation set. This difference could result from the fact that the ToxCast™ data, based on a model with inputs from 18 different assays, were used by most of the models as a training set, which we presume reduces the uncertainty. This is in contrast to the literature data, where the number of sources per chemical varied from one to a few hundreds. When only the subset of the evaluation set with more than six literature sources per chemical was considered, a large increase in the sensitivity was noticed (0.23 to 0.85).

**Table 4:** Confusion matrices of categorical *consensus* predictions for binding.

Observed/predicted	ToxCast^™^ data predicted actives	ToxCast^™^ data predicted inactives	Literature evaluation set (all: 7,283) predicted actives	Literature evaluation set (all: 7,283) predicted inactives
Observed actives	76	13	467	1,515
Observed inactives	25	1,415	268	5,033

**Table 5:** Statistics of categorical *consensus* predictions for binding on ToxCast™ and literature data.

Statistics/used data	ToxCast^™ ^data	Literature evaluation set (all: 7,283)	Literature evaluation set (> 6 sources: 1,257)
Sensitivity	0.85	0.23	0.85
Specificity	0.98	0.95	0.97
Balanced accuracy	0.92	0.59	0.91
The literature data with more than six sources represents the most consistent part of the evaluation set.			

**Figure 1:**
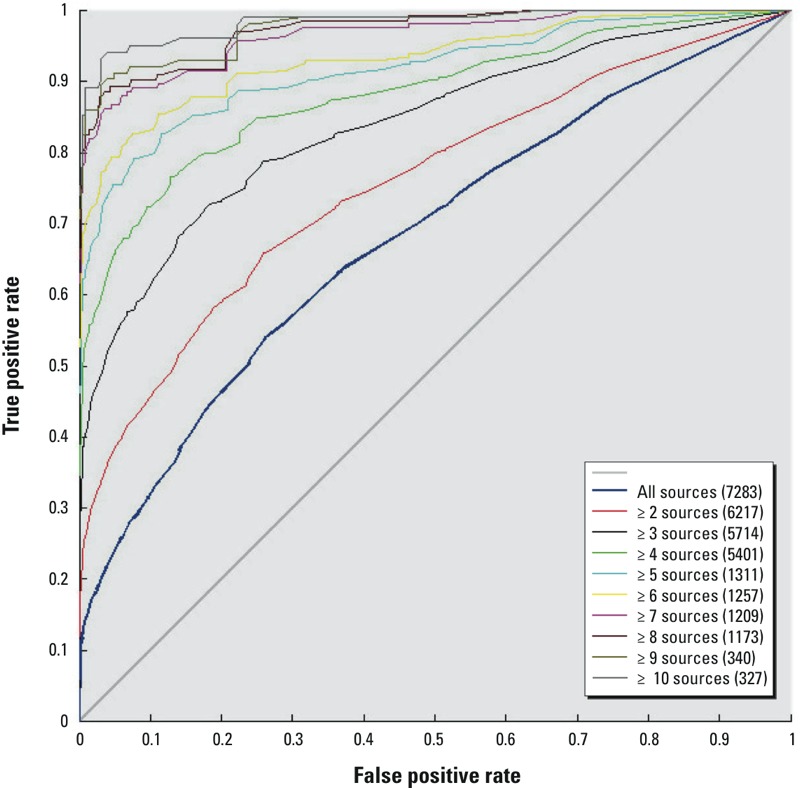
ROC curves of the categorical corrected consensus predictions for binding evaluated against different sets of the evaluation set with variable numbers of literature sources. The number of available chemicals in the evaluation set (between brackets) decreased with higher numbers of literature sources. The true and false positive rates are determined based on the number of actives in the different sets of the evaluation set.

To better understand the effect of the number of sources on the classification accuracy, ROC (receiver operating characteristic) curves were made using the fraction of the binding models in each class as a threshold for the classification predictions and increasing the number of literature sources of the evaluation set. The ROC plot shows an improvement of the classification accuracy of the *consensus* model as the number of sources increases (Figure 1). Note that the same level of consistency (i.e., 80%) was required to merge the sources regardless of the number of sources (see rule 1 in the “Evaluation set for categorical models” section). This could lead to the conclusion that the low classification accuracy on the full literature data is not because of a lack of accuracy of the *consensus* predictions, but rather to noise and experimental uncertainty in the literature data. We assume that the high number of false negatives in the confusion matrix of Table 4 is caused by false positives in the full literature data for chemicals tested only a small number of times. Thus, by considering a higher number of sources (i.e., six), the number of false positives is reduced from the evaluation set and so the number of predicted false negatives decreased. This is in agreement with what was observed in the literature (Steinmetz et al. 2014).

### Corrections to the *Consensus* Model

The first step of *consensus* modeling was conducted in an independent way for the categorical and continuous models on binding, agonist, and antagonist predictions. This led to a number of inconsistencies because some chemicals were predicted as active in categorical predictions but inactive in quantitative and vice versa. In addition, some chemicals were predicted as active agonists or antagonists but non-binders. To make all predictions more consistent, a number of corrections were applied on the first *consensus* predictions. Because the goal of this project was to help in a regulatory prioritization procedure, the modifications aimed to reduce the number of false negatives but without adding an excess of false positives. The rules that were followed to obtain the final *consensus* predictions are as follows:

If a chemical *i* is active in the categorical *consensus*, then it is also considered active in the quantitative *consensus*.
If a chemical *i* is active in the quantitative *consensus* and predicted as active by at least three categorical models, then it is also considered active in the categorical consensus.
If a chemical *i* is predicted active by less than three categorical models, then it is considered inactive also in quantitative *consensus*.

These three rules were applied on the agonist and antagonist *consensus* models first, then on the binding consensus. A fourth rule was added to establish consistency between agonist and antagonist *consensus* models and the binding *consensus* model.

If a chemical *i* is an active agonist or active antagonist, then it is considered as active in categorical binding *consensus,* and its potency level in the quantitative binding *consensus* is made equal to its potency level as agonist/antagonist.

**Figure 2:**
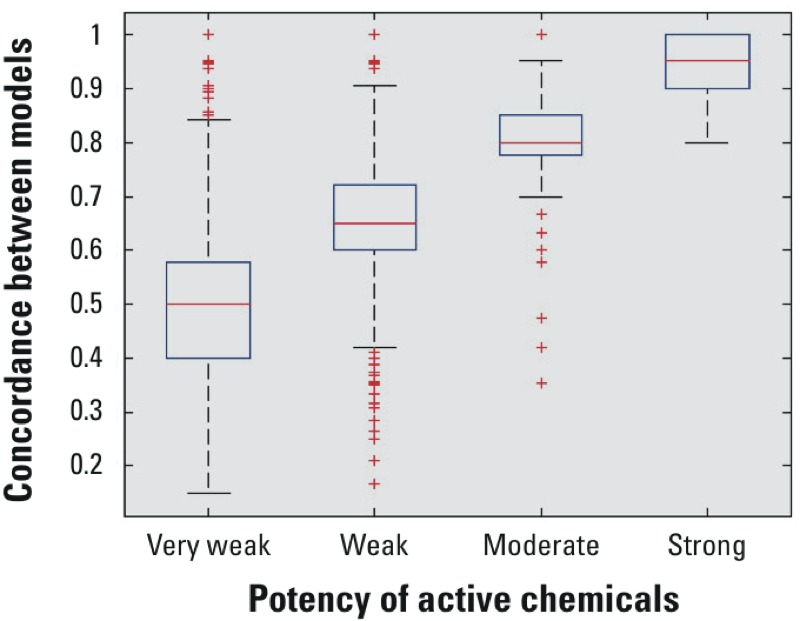
Box-plot of the positive class potency levels in the corrected quantitative *consensus* predictions for binding. The concordance between models is the fraction of the number of models that agrees on the prediction of a certain chemical. Boxes extend from the 25th to the 75th percentile, horizontal bars represent the median, whiskers indicate the 10th and 90th percentiles, and outliers are represented as points.

An analysis of variance in concordance in each potency level of the active chemicals in the continuous models (very weak, weak, moderate, and strong) is presented as a box-plot in Figure 2. Based on this figure, we noticed a correlation between the concordance of the categorical models and the potency level of active chemicals. This implies that models are more in agreement for strong actives and that the weaker a chemical is the more difficult it is to accurately predict. Therefore, the very weak chemicals are the main source of discordance among the different *in silico* models and also are the most uncertain experimentally. This relationship between positive concordance (agreement between models on predictions for active chemicals) and potency level for active chemicals can be used to set a quantitative prediction to the newly reclassified active chemicals using the previously mentioned rule 1 of the corrections applied to the consensus predictions. The following thresholds were considered for each potency level:

Strong: Concordance among models ≥ 0.9
Moderate: 0.75 ≤ Concordance among models < 0.9
Weak: 0.6 ≤ Concordance among models < 0.75
Very weak: Concordance among models < 0.6

After applying the four correction rules on consensus predictions, the total number of chemicals predicted as actives increased from 2,661 to 4,001, which corresponds to 12.3% of the total number of the prediction set (32,464). Table 6 shows the number of reclassified chemicals based on each one of the four correction rules applied to the consensus predictions. After this step, the predicted activity of several chemicals has changed. The structural information of chemicals and the predictions of the *consensus* model for the whole 32 k set are provided on the U.S. EPA ToxCast™ web site (see https://www3.epa.gov/research/COMPTOX/CERAPP_files.html, PredictionSet.zip) (U.S. EPA 2016).

**Table 6:** Number of chemicals reclassified after applying each one of the four prediction correction rules.

Rule used for each class	Rule 1			Rule 2			Rule 3			Rule 4
	Agonist	Antagonist	Binding	Agonist	Antagonist	Binding	Agonist	Antagonist	Binding	Binding
Number of chemicals	1,288	2,760	1,587	217	14	344	145	161	38	966
Rule 1: Chemicals that changed from inactive to active in the quantitative consensus based on the categorical *consensus*. Rule 2: Chemicals that changed from inactive to active in the categorical consensus based on the quantitative consensus. Rule 3: Chemicals that changed from active to inactive in the quantitative consensus based on the predictions of the categorical consensus. Rule 4: Chemicals that changed from inactive to active in the categorical binding consensus based on their agonist and antagonist activity in the categorical consensus.										

The confusion matrices and statistics for the binding categorical *consensus* model after modifications evaluated on ToxCast™ data and the literature data are presented in Table 7 and Table 8, respectively. The effect of the number of sources on the classification accuracy of the *consensus* model is illustrated by a bar plot in Figure S2. This figure shows an improvement of sensitivity with the increase in the number of literature sources in the evaluation set (from ~ 0.3 with at least one source to > 0.6 with six sources and more). This is translated into an increase in BA, whereas specificity is almost constant (~ 0.9) because of the high number of inactives compared to active compounds.

**Table 7:** Confusion matrices of the modified categorical *consensus* predictions for binding.

Observed/predicted	ToxCast^™^ data predicted actives	ToxCast^™^ data predicted inactives	Literature evaluation set (All: 7,283) predicted actives	Literature evaluation set (All: 7,283) predicted inactives
Observed actives	83	6	597	1,385
Observed inactives	40	1,400	463	4,838

**Table 8:** Statistics of the modified categorical *consensus* for binding predictions on ToxCast™ and literature data.

Statistics/used data	ToxCast^™^ data	Literature evaluation set (All: 7,283)	Literature evaluation set (> 6 Sources: 1,275)
Sensitivity	0.93	0.30	0.87
Specificity	0.97	0.91	0.94
Balanced accuracy	0.95	0.61	0.91

The results of this project and the ToxCast™ data used as the training set are published online in the EDSP21 dashboard, together with other structural and experimental assay information (see “Consensus CERAPP QSAR ER Model Predictions” under “Chemical Summary” tab on http://actor.epa.gov/edsp21) (U.S. EPA 2014c). A comparison of the single classification models to the *consensus* predictions for the whole 32 k set of chemicals is provided in Table S6. The calculations are done using the categorical consensus predictions as the “observed response.”

**Figure 3:**
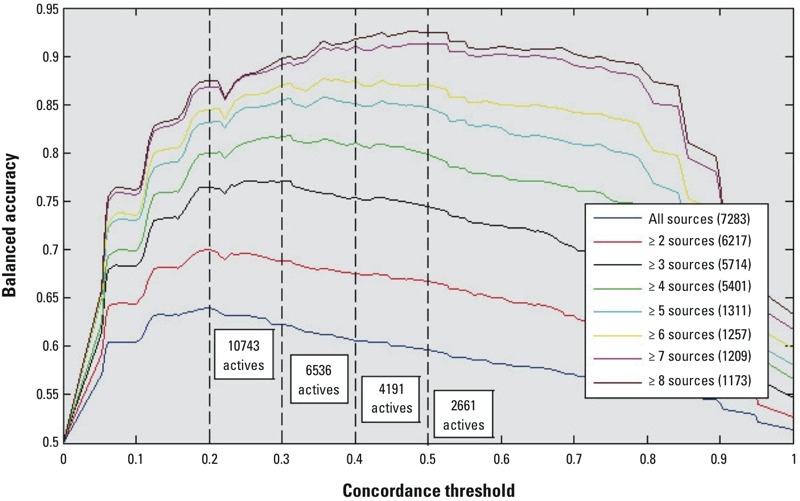
Variation of the balanced accuracy of the corrected categorical consensus predictions for binding with positive concordance (agreement between models on predictions for active chemicals) threshold at different numbers of literature sources.

For regulatory or prioritization purposes, one could use a looser definition of active (i.e., allow more disagreement among models) in order to further reduce the chance of false negatives. Figure 3 shows the number of chemicals that can be predicted as potential actives by the categorical consensus for binding using various positive concordance (agreement on actives between the included models) thresholds. When this threshold is set to 0.2, an additional 6,742 more chemicals can be added to the potential positives (this refers to the available binding models). This figure also shows the BA variations at different numbers of literature sources in the literature. Balanced accuracy increases as the concordance threshold increases from 0 to 0.2 because sensitivity increases (false negatives decrease) as the number of chemicals classified as active increases. For chemicals with the highest data quality (seven or more sources), the BA curve reaches a plateau at concordance thresholds of 0.4–0.5, and the number of chemicals classified as active is consistent with the number of active chemicals predicted from our consensus model (*n* = 4,001.) However, higher concordance thresholds result in declining BA due to increasing numbers of false positive predictions (i.e., decreasing specificity).

## Conclusion

The collaborative efforts of the CERAPP participants resulted in *consensus* predictions of the ability of chemicals to interact with ER. Up to 48 separately developed categorical and continuous models were received from 17 research groups from the United States and Europe. Separate models were built for agonist, antagonist, and binding activity. The models were applied to a large collection of 32,464 chemical structures that approximate the human exposure universe (chemicals with potential human exposure). A KNIME workflow was developed to carefully curate the large collection of chemical structures to ensure consistency in model development and evaluation. Most of the models were trained using activities derived from a data set combining 18 *in vitro* assays from ToxCast™ probing various points of the ER pathway. Models were then evaluated using the ToxCast™ data plus a collection of ER *in vitro* data from the literature. After this process, categorical predictions were combined into a consensus to classify the chemicals into actives and inactives, while continuous predictions were combined to classify the actives into 4 different potency classes: very weak, weak, moderate, and strong.

One major observation was that most models had comparable performances, independent of the methods used, with a slight improvement for models with narrow ADs. A second and, perhaps, more important observation is that the most concordant predictions come from comparing the *consensus* of many models with a *consensus* of many literature sources. For instance, when comparing the *consensus* of the categorical binding models with the evaluation set from the literature for chemicals with seven or more sources, we achieve a balanced accuracy of about 90% (Table 8).

We propose several important conclusions from our results. First, there does not appear to be an optimal modeling approach (combination of descriptor set, feature selection, or machine learning algorithm) that will solve the QSAR/docking problem and achieve perfect prediction accuracies. Second, there are inherent limitations to the accuracy of the data being used to train QSAR and docking models. Our analysis of the literature data showed a disagreement in the reported activity of many chemicals. The sources of discrepancy include limits to the concentration ranges tested, true differential activity among tissue sources [e.g., the presence of selective ER modulators, SERMs (selective estrogen receptor modulators)], and a variety of experimental artifacts and errors. Figure 2 shows that the most consistent predictions are achieved for the most potent compounds, whereas weaker compounds are called inactive by some laboratories because these compounds were not tested at a high enough concentration. So chemicals with very weak activity would be more likely to be incorrectly classified as inactive than more potent chemicals. Therefore, 100% accuracy cannot be achieved due to these limitations in the experimental data used for training and evaluation. Figures 1 and 3 help to illustrate this point by showing that higher consistency in the experimental data is associated with an increase in the concordance among model predictions. But this comes at the cost of excluding parts of the experimental data. So, just as every model has limitations, every *in vitro* assay also has inherent variability in its results.

The major purpose of this study was to identify potential ER actives out of the large universe of chemicals to which humans potentially are exposed using a *consensus* of *in silico* models to overcome the limitations of single models. Most of the chemicals in this collection were predicted to be negatives, with a high agreement among the individual models. The disagreement was the highest for chemicals with weak activity (Figure 2). This disagreement is driven by the difficulties in experimentally assessing the activity of these weak chemicals. In total, the consensus predicted 4,001 chemicals as actives. The testing of these active chemicals will be prioritized from the most potent to the least according to the continuous model *consensus* predictions. There are 6,742 more chemicals that 20–50% of the models predicted to be positive, which could also be candidates for follow-up analyses. Although this large number of chemicals (~ 10,000 in total) appears to be a daunting set to evaluate experimentally, this is equivalent in size to the current Tox21 library already being tested for activity in ER and many other targets.

In summary, this project demonstrates the feasibility of screening a large and toxicologically relevant library of chemical structures in an extensive battery of QSAR and docking models to meet important goals in human and environmental health. ER provides a good initial case because of the ready availability of experimental data and pre-existing models. However, through the ToxCast™ and Tox21 programs, and through other large scale data-integration projects, equivalently large data sets will become available for other multiple targets of environmental importance.

## Supplemental Material

(404 KB) PDF
