# Supplementary material for: CERAPP: Collaborative Estrogen Receptor Activity Prediction Project
Source: Environ Health Perspect. 2016 Feb 23;124(7):1023–33. doi: 10.1289/ehp.1510267 (PMC4937869; doi:10.1289/ehp.1510267)
Supplement: (404 KB) PDF [file ehp.1510267.s001.acco.pdf]

Environ Health Perspect
DOI: 10.1289/ehp.1510267

Note to readers with disabilities: *EHP* strives to ensure that all journal content is accessible to all readers. However, some figures and Supplemental Material published in *EHP* articles may not conform to 508 standards due to the complexity of the information being presented. If you need assistance accessing journal content, please contact ehp508@niehs.nih.gov. Our staff will work with you to assess and meet your accessibility needs within 3 working days.

Supplemental Material

CERAPP: Collaborative Estrogen Receptor Activity Prediction Project

Kamel Mansouri, Ahmed Abdelaziz, Aleksandra Rybacka, Alessandra Roncaglioni, Alexander Tropsha, Alexandre Varnek, Alexey Zakharov, Andrew Worth, Ann M. Richard, Christopher M. Grulke, Daniela Trisciuzzi, Denis Fourches, Dragos Horvath, Emilio Benfenati, Eugene Muratov, Eva Bay Wedebye, Francesca Grisoni, Giuseppe F. Mangiatordi, Giuseppina M. Incisivo, Huixiao Hong, Hui W. Ng, Igor V. Tetko, Ilya Balabin, Jayaram Kancherla, Jie Shen, Julien Burton, Marc Nicklaus, Matteo Cassotti, Nikolai G. Nikolov, Orazio Nicolotti, Patrik L. Andersson, Qingda Zang, Regina Politi, Richard D. Beger, Roberto Todeschini, Ruili Huang, Sherif Farag, Sine A. Rosenberg, Svetoslav Slavov, Xin Hu, and Richard S. Judson

Table of Contents

Table S1: Information on participating groups
Table S2: Project plan and tasks accomplished in each step
Table S3: Statistics and scores of the categorical models of the different research groups in alphabetic order.
Table S4: Sensitivity and specificity of the different evaluation steps for categorical models
Table S5: statistics and scores of agonist, antagonist and binding continuous models based on the five potency classes defined using the reference chemicals.

Table S6: Participant single categorical models compared to agonist antagonist and binding categorical *consensus* predictions (as the "observed response") after the 4 correction rules were applied. It shows the number of predicted chemicals (out of the total 32k prediction set), the number of actives (out of the 4001 actives predicted by the consensus categorical model) and summary statistics based on the consensus categorical predictions.

Figure S1: Number of chemicals by positive concordance (agreement on actives between the included models) of the categorical binding models on the active compounds of the prediction set (32k). The positive concordance is the fraction of models predicting a certain chemical as active by the total number of models providing a prediction for that chemical.

Figure S2: Bar plot of the corrected categorical *consensus* for binding accuracy with variable number of literature sources from the evaluation set. The y –axis, depending on the color of the bar from the legend, represents the balanced accuracy, sensitivity or specificity.

Table S1: Information on participating groups

Abbreviation	Name of the group	Institution
DTU	National Food Institute/Division of Toxicology and Risk Assessment	Technical University of Denmark, Denmark
EPA_NCCT	National Center for Computational Toxicology	U.S. Environmental Protection Agency, USA
FDA_NCTR_DBB	National Center for Toxicological Research/Division of Bioinformatics and Biostatistics	U.S. Food and Drug Administration, USA
FDA_NCTR_DSB	National Center for Toxicological Research/Division of Systems Biology	U.S. Food and Drug Administration, USA
ILS&EPA_NCCT	ILS Inc & National Center for Computational Toxicology	ILS Inc & U.S. EPA, USA
IRCCS	Environmental Chemistry and Toxicology Laboratory	Istituto di Ricerche Farmacologiche "Mario Negri", Italy
JRC_Ispra	Institute for Health & Consumer Protection (IHCP)	Joint Research Centre of the European Commission in Ispra, Italy
LockheedMartin&EPA	High Performance Computing	Lockheed Martin & U.S. EPA, USA
NIH_NCATS	National Center for Advancing Translational Sciences	National Institutes of Health, USA
NIH_NCI	National Cancer Institute	National Institutes of Health, USA
OCHEM	Cheminformatics group	Institute of Structural Biology Helmholtz Zentrum Muenchen, Germany
RIFM	Computational Toxicology	Research Institute for Fragrance Materials, Inc, USA
Umeå	Chemistry Department	Umeå University, Sweden
UNC_MML	Laboratory for Molecular Modeling	University of North Carolina, USA
UNIBA	Department of Pharmacy-Drug Sciences	University of Bari, Italy
UNIMIB	Milano Chemometrics and QSAR Research Group	University of Milano-Bicocca, Italy
UNISTRA	Laboratoire de Chemoinformatique	University of Strasbourg, France

Table S2: Project plan and tasks accomplished in each step

Steps	Tasks	Research Groups
**Step 1:** Chemical structure curation	- Merge chemical structures from different sources<br>- Design and document a workflow for structure curation<br>- Deliver the QSAR-ready training set and prediction set	-EPA_NCCT<br>-UNC_MML<br>-DTU
**Step 2:** Experimental data preparation from literature for models validation	- Collect experimental data from literature for the evaluation set<br>- Combine the different assays / results into a unique score<br>- Define a strategy to evaluate the individual models	-EPA_NCCT<br>-Umeå<br>-NIH_NCATS<br>-FDA_NCTR_DBB<br>- OCHEM
**Step 3:** Modeling and predictions	- Train/refine the models based on the training set<br>- Make predictions using the individual models<br>- Deliver predictions and applicability domains (AD) for evaluation	All groups
**Step 4:** Model evaluation	- Analyze the training and evaluation datasets<br>- Evaluate the predictions of each model separately	-EPA_NCCT
**Step 5:** *Consensus* strategy development	- Develop strategies to create *consensus* scores<br>- Define a score for each model based on the evaluation step<br>- Define a weighting scheme based on scores	- EPA_NCCT (Discussed with all groups)
**Step 6:** *Consensus* modeling (and validation)	- Combine the predictions based on the weighting scheme<br>- (Validate the *consensus* model using an experimental evaluation set) if ready?	-EPA_NCCT

Table S3: Statistics and scores of the categorical models of the different research groups in alphabetic order.

**Agonist models**	ToxCast data	BA ToxCast	Literature data	BA Literature	Multi. Src.	BA Multi Src	Literature non _vw	BA non_vw	In AD	BA AD	All filters.	BA All filters	% predicted	score_1	score_2
DTU_METI	737	0.81	2719	0.77	2662	0.81	2705	0.77	2493	0.79	2432	0.85	41.41	0.38	0.83
DTU_TOXCAST	1447	0.89	5343	0.79	5279	0.82	5328	0.79	4679	0.81	4618	0.85	84.14	0.78	0.87
FDA_NCTR_DBB	1529	0.99	6319	0.75	6251	0.78	6300	0.76	6319	0.75	6232	0.79	100	0.92	0.89
LockheedMartin_1	1529	0.82	6319	0.69	6251	0.72	6300	0.70	1684	0.80	1667	0.84	100	0.78	0.83
LockheedMartin_2	1529	0.76	6319	0.67	6251	0.69	6300	0.68	1684	0.75	1667	0.78	100	0.75	0.77
JRC_Ispra	1465	0.81	5995	0.77	5929	0.81	5976	0.78	5995	0.77	5910	0.81	94.88	0.82	0.81
NIH_NCI_GUSAR	1529	0.99	6319	0.80	6251	0.82	6300	0.81	6277	0.80	6191	0.84	99.97	0.93	0.91
NIH_NCI_PASS	1465	0.87	5995	0.76	5929	0.78	5976	0.77	5995	0.76	5910	0.79	94.87	0.84	0.83
OCHEM	1516	0.91	6200	0.80	6134	0.82	6182	0.81	6200	0.80	6116	0.83	97.75	0.89	0.87
UNIBA	755	0.85	2899	0.82	2875	0.83	2888	0.82	2899	0.82	2864	0.84	47.51	0.42	0.84
UNISTRA	1529	0.85	6319	0.73	6251	0.76	6300	0.74	5366	0.69	5298	0.73	100	0.85	0.79
**Antagonist models**															
FDA_NCTR_DBB	1529	0.99	6539	0.55	6538	0.55	6463	0.56	6539	0.55	6462	0.56	100	0.85	0.78
LockheedMartin_1	1529	0.89	6539	0.52	6538	0.52	6463	0.52	6539	0.52	6462	0.52	100	0.80	0.70
LockheedMartin_2	1529	0.72	6539	0.51	6538	0.51	6463	0.51	6539	0.51	6462	0.51	100	0.74	0.62
JRC_Ispra	1465	0.75	6186	0.55	6185	0.55	6115	0.57	6186	0.55	6114	0.56	94.88	0.73	0.66
NIH_NCI_GUSAR	1529	1.00	6539	0.54	6538	0.54	6463	0.55	6509	0.54	6433	0.55	99.97	0.85	0.77
NIH_NCI_PASS	1465	0.79	6186	0.49	6185	0.49	6115	0.49	6186	0.49	6114	0.49	94.87	0.72	0.64
OCHEM	1516	0.72	6431	0.63	6430	0.63	6356	0.65	6431	0.63	6355	0.65	97.75	0.77	0.69
UNIBA	434	0.92	1723	0.63	1722	0.63	1706	0.65	1723	0.63	1705	0.65	25.98	0.23	0.78

**Binding models**															
DTU_METI	873	0.82	3840	0.64	3243	0.71	3482	0.68	3306	0.66	2695	0.78	49.48	0.43	0.80
EPA_NCCT	1529	0.87	7283	0.57	6217	0.63	6598	0.60	6383	0.58	5275	0.69	100	0.82	0.78
FDA_NCTR_DBB	1529	0.99	7283	0.60	6217	0.66	6598	0.63	7283	0.60	5991	0.68	100	0.87	0.84
FDA_NCTR_DSB	1019	0.80	534	0.62	450	0.65	492	0.64	534	0.62	431	0.66	0.62	-	0.69
ILS_EPA	1506	0.84	7068	0.66	6031	0.73	6404	0.69	7068	0.66	5814	0.75	96.47	0.82	0.79
IRCCS_CART	1529	0.80	7280	0.61	6214	0.67	6595	0.63	4326	0.62	3620	0.75	99.99	0.78	0.77
IRCCS_Ruleset	1383	0.91	6603	0.56	5621	0.61	5978	0.58	6603	0.56	5416	0.62	89.20	0.75	0.77
JRC_Ispra	1465	0.82	6900	0.58	5885	0.64	6250	0.62	6900	0.58	5672	0.67	94.88	0.77	0.74
LockheedMartin_1	1529	0.83	7283	0.55	6217	0.59	6598	0.57	1977	0.56	1539	0.66	100	0.75	0.75
LockheedMartin_2	1529	0.76	7283	0.54	6217	0.58	6598	0.56	1977	0.56	1539	0.64	100	0.72	0.70
NIH_NCATS	1528	0.69	7271	0.59	6207	0.63	6587	0.61	7271	0.59	5981	0.65	99.13	0.77	0.67
NIH_NCI_GUSAR	1529	0.99	7283	0.61	6217	0.66	6598	0.64	7235	0.61	5951	0.69	99.97	0.88	0.84
NIH_NCI_PASS	1465	0.86	6900	0.58	5885	0.63	6250	0.61	6900	0.58	5672	0.66	94.87	0.78	0.76
OCHEM	1512	0.89	7123	0.62	6081	0.69	6455	0.65	7123	0.62	5860	0.72	97.43	0.83	0.80
RIFM	1529	0.73	7283	0.58	6217	0.64	6598	0.59	7283	0.58	5991	0.65	100	0.78	0.69
Umeå	1529	0.82	7280	0.61	6215	0.68	6596	0.64	7280	0.61	5989	0.70	99.89	0.82	0.76
UNC_MML	1529	0.80	7283	0.59	6217	0.63	6598	0.61	7283	0.59	5991	0.65	100	0.80	0.73
UNIBA	750	0.86	3259	0.62	2853	0.69	2963	0.67	3259	0.62	2753	0.73	46.75	0.40	0.80
UNIMIB_1	1529	0.76	7283	0.55	6217	0.57	6598	0.56	7283	0.55	5991	0.59	100	0.77	0.68
UNIMIB_2	531	0.98	2780	0.62	2348	0.69	2475	0.65	2780	0.62	2241	0.71	36.44	0.32	0.85
UNISTRA	1529	0.86	7283	0.57	6217	0.62	6598	0.60	5800	0.54	4755	0.60	100	0.80	0.73

The number of chemicals used for the evaluation of each model for the different steps is also provided. ToxCast data: predictions overlapping with the 1529 ToxCast chemicals. BA: balanced accuracy; Multi. Src: number of compounds in the literature data with multiple literature sources; non_vw: number of compounds in the literature data that are not very weak binders; In AD: number of compounds inside the applicability domain; All filters: remaining compounds after removing the chemicals with single source, very weak and outside the AD, % predicted: the fraction of the predicted chemicals by a certain model out of the total 32k list, score_1 and score_2 are scoring functions calculated using equations 1 and 2, respectively.

Table S4: Sensitivity and specificity of the different evaluation steps for categorical models

**agonist models**	SN ToxCast	SP ToxCast	SN All literature	SP All literature	SN multSrc Literature	multSrc SP Literature	SN no_VW literature	SP no_VW literature	SN in_AD Literature	SP in_AD Literature	SN allPar Literature	SP allPar_ Literature
DTU_METI	0.689	0.922	0.624	0.918	0.705	0.919	0.630	0.918	0.644	0.943	0.751	0.944
DTU_TOXCAST	0.875	0.904	0.673	0.898	0.738	0.898	0.689	0.898	0.678	0.932	0.764	0.932
FDA_NCTR_DBB	1.000	0.986	0.560	0.949	0.619	0.950	0.565	0.949	0.560	0.949	0.629	0.950
JRC_Ispra	0.725	0.890	0.640	0.910	0.706	0.911	0.646	0.910	0.640	0.910	0.719	0.911
LockheedMartin_1	0.650	0.999	0.400	0.990	0.455	0.990	0.417	0.990	0.604	0.997	0.681	0.998
LockheedMartin_2	0.525	0.990	0.357	0.985	0.402	0.985	0.375	0.985	0.509	0.992	0.571	0.992
NIH_NCI_GUSAR	1.000	0.981	0.737	0.865	0.783	0.865	0.752	0.865	0.741	0.865	0.808	0.865
NIH_NCI_PASS	0.988	0.747	0.767	0.758	0.801	0.759	0.778	0.758	0.767	0.758	0.817	0.759
OCHEM	0.949	0.866	0.748	0.857	0.792	0.858	0.755	0.857	0.748	0.857	0.804	0.858
UNIBA	0.755	0.936	0.760	0.873	0.792	0.873	0.774	0.873	0.760	0.873	0.811	0.873
UNISTRA	0.725	0.977	0.503	0.963	0.566	0.963	0.514	0.963	0.415	0.962	0.492	0.962
**antagonist models**												
FDA_NCTR_DBB	1.000	0.978	0.113	0.984	0.110	0.984	0.144	0.984	0.113	0.984	0.140	0.984
JRC_Ispra	0.667	0.827	0.263	0.842	0.260	0.842	0.290	0.842	0.263	0.842	0.286	0.842
LockheedMartin_1	0.778	1.000	0.039	0.997	0.035	0.997	0.048	0.997	0.039	0.997	0.043	0.997
LockheedMartin_2	0.444	0.997	0.028	0.996	0.025	0.996	0.034	0.996	0.028	0.996	0.029	0.996
NIH_NCI_GUSAR	1.000	0.992	0.204	0.878	0.201	0.878	0.221	0.878	0.196	0.880	0.211	0.880
NIH_NCI_PASS	1.000	0.586	0.393	0.591	0.390	0.591	0.398	0.591	0.393	0.591	0.394	0.591
OCHEM	0.667	0.774	0.479	0.782	0.477	0.782	0.522	0.782	0.479	0.782	0.519	0.782
UNIBA	1.000	0.837	0.442	0.819	0.434	0.819	0.483	0.819	0.442	0.819	0.475	0.819

**binding models**												
DTU_METI	0.805	0.828	0.428	0.848	0.579	0.849	0.503	0.848	0.421	0.890	0.665	0.891
EPA_NCCT	0.865	0.877	0.297	0.848	0.418	0.851	0.352	0.848	0.309	0.860	0.517	0.863
FDA_NCTR_DBB	1.000	0.977	0.262	0.935	0.374	0.938	0.318	0.935	0.262	0.935	0.425	0.938
FDA_NCTR_DSB	0.820	0.780	0.706	0.528	0.776	0.521	0.758	0.528	0.706	0.528	0.801	0.521
ILS_EPA	0.943	0.863	0.525	0.842	0.665	0.846	0.585	0.842	0.525	0.842	0.696	0.795
IRCCS_CART	0.708	0.738	0.314	0.793	0.437	0.795	0.371	0.793	0.302	0.793	0.557	0.939
IRCCS_Ruleset	0.940	0.883	0.253	0.898	0.344	0.899	0.292	0.898	0.253	0.938	0.383	0.866
JRC_Ispra	0.843	0.883	0.370	0.863	0.485	0.866	0.439	0.863	0.370	0.863	0.536	0.804
LockheedMartin_1	0.663	0.795	0.109	0.800	0.189	0.804	0.153	0.800	0.132	0.800	0.326	0.997
LockheedMartin_2	0.539	1.000	0.109	0.985	0.180	0.985	0.149	0.985	0.126	0.995	0.295	0.988
NIH_NCATS	0.517	0.990	0.326	0.979	0.410	0.979	0.366	0.979	0.326	0.987	0.451	0.854
NIH_NCI_GUSAR	1.000	0.866	0.381	0.853	0.483	0.854	0.439	0.853	0.382	0.853	0.546	0.837
NIH_NCI_PASS	0.978	0.983	0.418	0.835	0.518	0.838	0.472	0.835	0.418	0.834	0.565	0.747
OCHEM	0.920	0.741	0.405	0.741	0.538	0.747	0.467	0.741	0.405	0.741	0.587	0.846
RIFM	0.719	0.744	0.368	0.797	0.478	0.799	0.392	0.797	0.368	0.797	0.498	0.799
Umeå	0.730	0.911	0.312	0.904	0.449	0.904	0.370	0.904	0.312	0.904	0.500	0.904
UNC_MML	0.775	0.818	0.383	0.792	0.469	0.792	0.427	0.792	0.383	0.792	0.517	0.792
UNIBA	0.774	0.948	0.335	0.900	0.487	0.900	0.442	0.900	0.335	0.900	0.563	0.900
UNIMIB_1	0.865	0.658	0.365	0.726	0.417	0.730	0.400	0.726	0.365	0.726	0.449	0.730
UNIMIB_2	1.000	0.969	0.483	0.759	0.609	0.765	0.533	0.759	0.483	0.759	0.658	0.765
UNISTRA	0.730	0.981	0.182	0.955	0.286	0.957	0.236	0.955	0.109	0.969	0.222	0.972

SN: sensitivity; SP: specificity. Multsrc: number of compounds in the literature data with multiple literature sources; no_VW: not very weak binders in the literature data; in_AD: compounds inside the AD; AllPar: remaining compounds after removing the chemicals with single source, very weak and outside the AD

Table S5: statistics and scores of agonist, antagonist and binding continuous models based on the five potency classes defined using the reference chemicals.

**Agonist models**	ToxCast _data	BA ToxCast	Literature data	BA literature	Multi. Src	BA Multi Src	Literature non _vw	BA non_vw	All filters	BA All filters	predicted %	score	weights
LockheedMartin_1	1529	0.771	6163	0.504	6163	0.504	6144	0.500	6144	0.500	100	0.721	0.370
LockheedMartin_2	1529	0.686	6163	0.515	6163	0.515	6144	0.503	6144	0.503	100	0.577	0.295
UNISTRA	1529	0.703	6163	0.507	6163	0.507	6144	0.501	6144	0.501	100	0.654	0.335
**Antagonist models**													
LockheedMartin_1	1529	0.930	6505	0.500	6504	0.500	6429	0.501	6428	0.501	100	0.786	0.661
LockheedMartin_2	1529	0.544	6505	0.499	6504	0.499	6429	0.500	6428	0.500	100	0.403	0.339
**Binding models**													
LockheedMartin_1	1529	0.798	6770	0.504	5887	0.502	6085	0.502	5681	0.982	100	0.733	0.373
LockheedMartin_2	1529	0.673	6770	0.502	5887	0.500	6085	0.501	5681	0.979	100	0.581	0.296
UNISTRA	1529	0.688	6770	0.499	5887	0.498	6085	0.500	5681	0.980	100	0.649	0.331

The number of chemicals used for the evaluation of each model for the different steps is also provided. ToxCast data: predictions overlapping with the 1529 ToxCast chemicals. BA: balanced accuracy; Multi. Src: number of compounds in the literature data with multiple literature sources; non_vw: number of compounds in the literature data that are not very weak binders; All filters: remaining compounds after removing the chemicals with single source, very weak and outside the AD, % predicted: the fraction of the predicted chemicals by a certain model out of the total 32k list, score is a scoring function calculated using equation 2

Table S6: Participant single categorical models compared to agonist antagonist and binding categorical *consensus* predictions (as the "observed response") after the 4 correction rules were applied. It shows the number of predicted chemicals (out of the total 32k prediction set), the number of actives (out of the 4001 actives predicted by the consensus categorical model) and summary statistics based on the consensus categorical predictions.

**Agonist models**	Predicted	Actives	Sensitivity	Specificity	Balanced accuracy
DTU_METI	13442	1575	0.47	0.95	0.71
DTU_TOXCAST	27316	3515	0.80	0.93	0.87
FDA_NCTR_DBB	32464	2506	0.57	0.96	0.77
JRC_Ispra	30801	3549	0.72	0.94	0.83
LockheedMartin_1	32464	719	0.26	~1	0.63
LockheedMartin_2	32464	829	0.30	~1	0.65
NIH_NCI_GUSAR	32455	6410	0.90	0.86	0.88
NIH_NCI_PASS	30800	9051	0.94	0.76	0.85
OCHEM	31736	5867	0.94	0.88	0.91
UNIBA	15426	1906	0.72	0.91	0.81
UNISTRA	32464	1855	0.52	0.98	0.75
**Antagonist models**					
FDA_NCTR_DBB	32464	691	0.08	0.98	0.53
JRC_Ispra	30801	4967	0.71	0.89	0.80
LockheedMartin_1	32464	101	0.01	~1	~0.5
LockheedMartin_2	32464	117	~0	~1	~0.5
NIH_NCI_GUSAR	32455	4906	0.30	0.86	0.58
NIH_NCI_PASS	30800	13197	0.74	0.60	0.67
OCHEM	31736	8586	0.56	0.76	0.66
UNIBA	8433	1519	0.81	0.88	0.85
**Binding models**					
DTU_METI	16063	3952	0.55	0.83	0.69
EPA_NCCT	32463	5360	0.66	0.90	0.78
FDA_NCTR_DBB	32464	3070	0.56	0.97	0.77
FDA_NCTR_DSB	2008	1073	0.59	0.61	0.60
ILS_EPA	31318	8335	0.64	0.79	0.71
IRCCS_CART	32442	5170	0.48	0.88	0.68
IRCCS_Ruleset	28958	4387	0.51	0.9	0.71
JRC_Ispra	30801	7236	0.77	0.84	0.81
LockheedMartin_1	32464	815	0.19	~1	0.60
LockheedMartin_2	32464	944	0.22	~1	0.61
NIH_NCATS	32184	6006	0.51	0.86	0.68
NIH_NCI_GUSAR	32455	7563	0.88	0.86	0.87
NIH_NCI_PASS	30800	9343	0.86	0.78	0.82
OCHEM	31629	6429	0.91	0.90	0.91
RIFM	32463	8690	0.54	0.77	0.66
Umeå	32430	4405	0.38	0.90	0.64
UNC_MML	32464	7179	0.5	0.82	0.66
UNIBA	15178	1658	0.59	0.94	0.76
UNIMIB_1	32464	9922	0.68	0.75	0.71
UNIMIB_2	11832	3586	0.90	0.81	0.85
UNISTRA	32464	2003	0.39	0.98	0.68

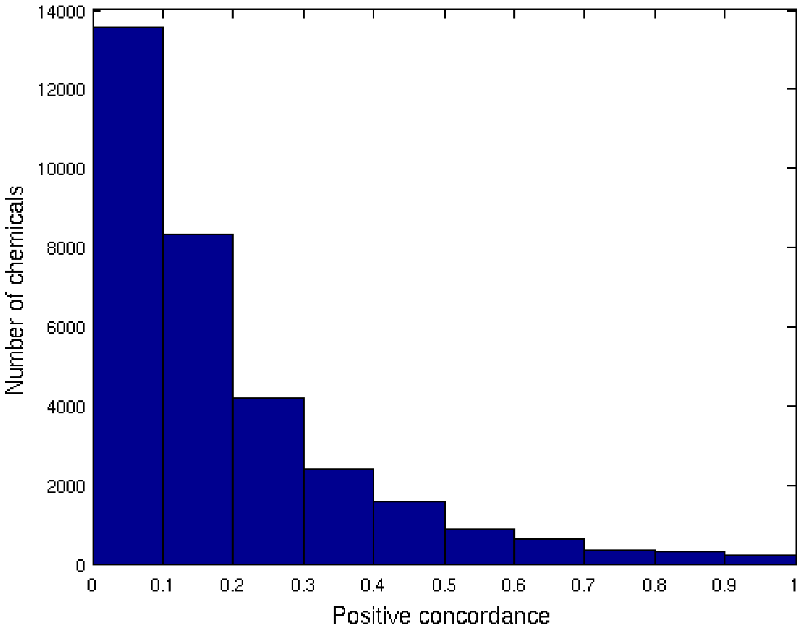

Figure S1: Number of chemicals by positive concordance (agreement on actives between the included models) of the categorical binding models on the active compounds of the prediction set (32k). The positive concordance is the fraction of models predicting a certain chemical as active by the total number of models providing a prediction for that chemical.

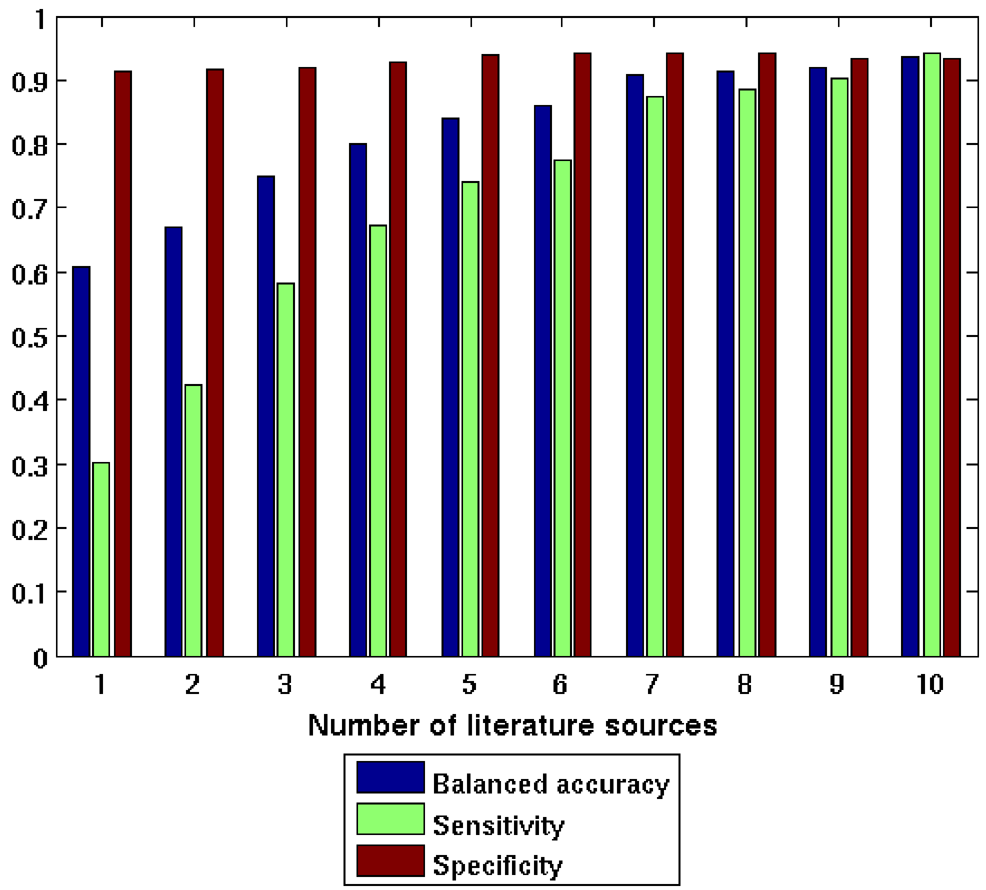

Figure S2: Bar plot of the corrected categorical *consensus* for binding accuracy with variable number of literature sources from the evaluation set. The y –axis, depending on the color of the bar from the legend, represents the balanced accuracy, sensitivity or specificity.
